# Nanobodies and Nanobody-Based Human Heavy Chain Antibodies As Antitumor Therapeutics

**DOI:** 10.3389/fimmu.2017.01603

**Published:** 2017-11-22

**Authors:** Peter Bannas, Julia Hambach, Friedrich Koch-Nolte

**Affiliations:** ^1^Department of Diagnostic and Interventional Radiology and Nuclear Medicine, Hamburg, Germany; ^2^Institute of Immunology, University Medical Center Hamburg-Eppendorf, Hamburg, Germany

**Keywords:** nanobodies, heavy chain antibodies, antitumor therapeutics, nanobody-conjugates, nanobody fusion proteins, sortagging of nanobodies

## Abstract

Monoclonal antibodies have revolutionized cancer therapy. However, delivery to tumor cells *in vivo* is hampered by the large size (150 kDa) of conventional antibodies. The minimal target recognition module of a conventional antibody is composed of two non-covalently associated variable domains (VH and VL). The proper orientation of these domains is mediated by their hydrophobic interface and is stabilized by their linkage to disulfide-linked constant domains (CH1 and CL). VH and VL domains can be fused *via* a genetic linker into a single-chain variable fragment (scFv). scFv modules in turn can be fused to one another, e.g., to generate a bispecific T-cell engager, or they can be fused in various orientations to antibody hinge and Fc domains to generate bi- and multispecific antibodies. However, the inherent hydrophobic interaction of VH and VL domains limits the stability and solubility of engineered antibodies, often causing aggregation and/or mispairing of V-domains. Nanobodies (15 kDa) and nanobody-based human heavy chain antibodies (75 kDa) can overcome these limitations. Camelids naturally produce antibodies composed only of heavy chains in which the target recognition module is composed of a single variable domain (VHH or Nb). Advantageous features of nanobodies include their small size, high solubility, high stability, and excellent tissue penetration *in vivo*. Nanobodies can readily be linked genetically to Fc-domains, other nanobodies, peptide tags, or toxins and can be conjugated chemically at a specific site to drugs, radionuclides, photosensitizers, and nanoparticles. These properties make them particularly suited for specific and efficient targeting of tumors *in vivo*. Chimeric nanobody-heavy chain antibodies combine advantageous features of nanobodies and human Fc domains in about half the size of a conventional antibody. In this review, we discuss recent developments and perspectives for applications of nanobodies and nanobody-based human heavy chain antibodies as antitumor therapeutics.

## Introduction

Monoclonal antibodies (mAbs) and antibody-derived biologics are essential tools for cancer research and therapy (1, 2). Antibodies can be used to inhibit tumor cell proliferation and as targeting moieties of effector domains. Many mAbs directed against tumor cell surface proteins interfere with the function of their target proteins, e.g., by blocking signaling *via* a growth factor receptor or by inducing apoptosis. By opsonizing the tumor cell, antibodies can also mark tumor cells for attack by the complement system, NK cells and macrophages. Antibody engineering provides powerful technologies to improve antibody effector functions and to generate novel, bispecific biologics. The use of mAbs has revolutionized antitumor therapy, with impressive achievements in the treatment of both hematological malignancies and solid tumors (3).

However, certain inherent structural properties limit the applicability of mAbs and antibody-derived biologics for tumor therapy. The large size of mAbs (four polypeptide chains, 150 kD) can hamper access to tumor cells. Moreover, the nature of the antibody recognition module—a pair of variable domains non-covalently associated *via* a hydrophobic interface—poses obstacles to the development of bispecific biologics. These aspects illustrate the need for new antibody formats that provide the same binding specificity of mAbs but with better stability and *in vivo* pharmacodynamics.

The discovery of naturally occurring heavy chain antibodies (hcAbs, two polypeptide chains, 75 kD) containing a highly stable and soluble single antigen-binding V-domain—designated VHH or nanobody (15 kDa)—has opened the way for a new generation of antitumor therapeutics. Other excellent reviews describe the discovery and structure of nanobodies, their potential applications in oncology, infection, immunity, and other diseases (4–18). Here, we focus on the unique features of nanobodies and nanobody-based human heavy chain antibodies that underlie their huge potential as antitumor therapeutics. We provide insight into the current status, ongoing developments and future challenges toward successful implementation of nanobodies and nanobody-based human hcAbs as antitumor therapeutics.

## The Antigen-Binding Modules of Conventional and Heavy Chain Antibodies

**Figure 1:**
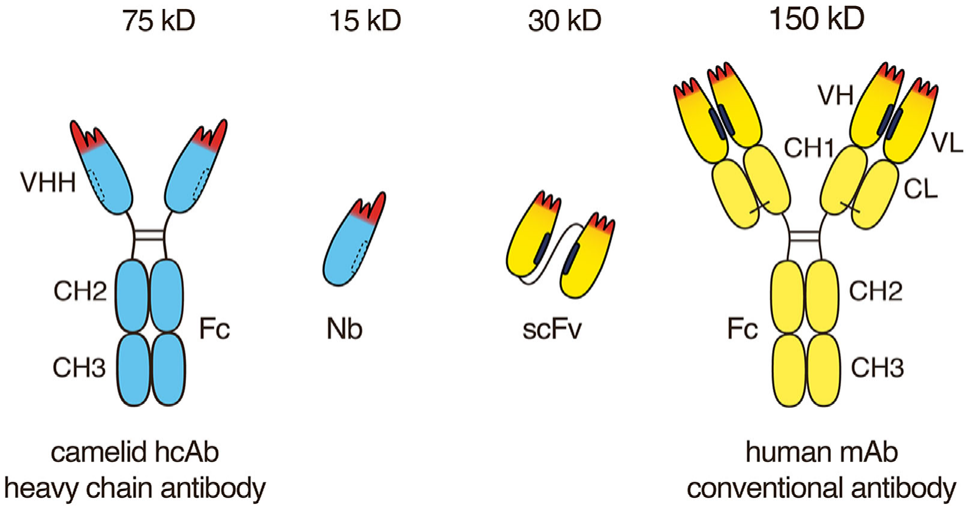
Advantageous features of camelid heavy chain antibodies. Heavy chain antibodies are composed of two heavy chains. The target-binding module is composed of a single VHH domain. A recombinant VHH domain, designated nanobody (Nb) is highly soluble and does not show any tendency to associate with other hydrophobic protein surfaces. Conventional antibodies are composed of two heavy and two light chains. The target-binding module is composed of two non-covalently associated variable domains VH and VL. In intact antibodies, the proper orientation of these domains is mediated by a hydrophobic interface (see Figure 1) and is further stabilized by the disulfide-linked CL and CH1 domains. A pair of VH and VL domains can be linked genetically into a single-chain variable fragment (scFv) in which the proper orientation of domains is mediated alone by the hydrophobic interface between the two V-domains.

**Figure 2:**
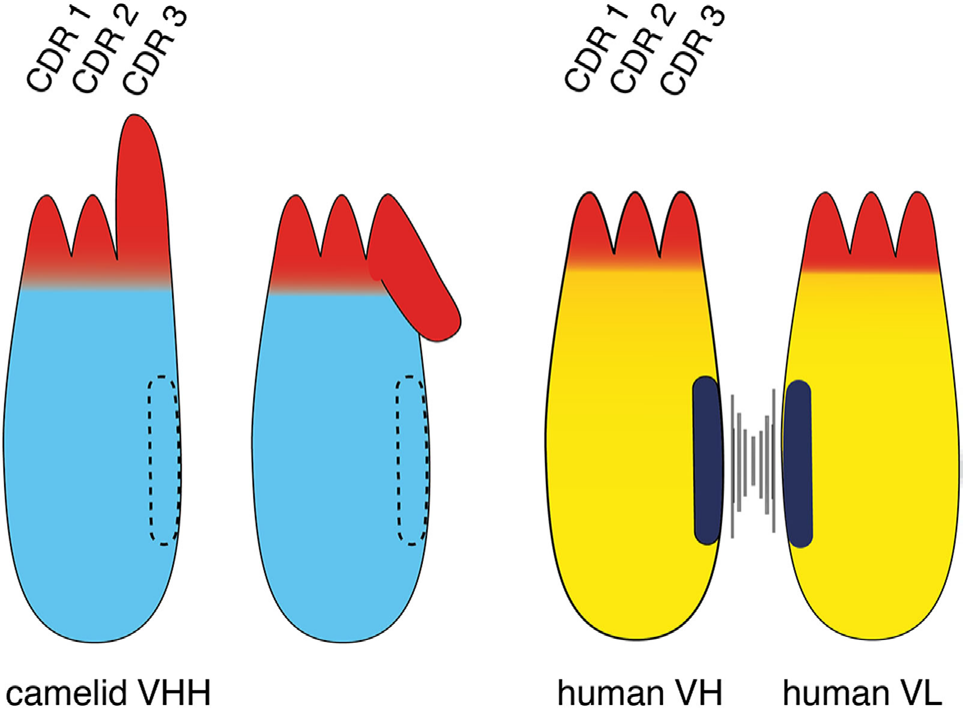
Comparison of the VHH domain (nanobody) of a camelid heavy chain antibody with its VH-counterpart of a conventional antibody. The three complementarity determining regions (CDRs) of the antigen-binding paratope are indicated in red, the framework region is indicated in cyan (camelid VHH) and yellow (human VH and VL). The CDR3 loop contributes most to the diversity and specificity of the paratope since its coding region is newly generated during B-cell development, i.e., by genetic fusion of a D element with flanking V and J elements and deletion or insertion of nucleotides at the junctions. The CDR3 loop of a camelid VHH typically is much longer than that of a human VH. A key distinguishing feature of a camelid VHH is that it binds its target with a single domain, whereas a human VH binds its target together with a non-covalently associated VL. A second distinguishing feature of a VHH is its entirely hydrophilic framework, whereas a VH domain contains a hydrophobic side facing the VL domain (indicated in black). This hydrophobic interface helps to maintain the proper orientation of the six CDR loops of the target-binding paratope. This hydrophobic interface accounts for the inherent stickiness of isolated VH domains, and for the propensity of “mispairing” of VH and VL domains in bispecific antibody (bsAb) constructs. The corresponding region in a VHH is hydrophilic (indicated by dashed lines), accounting for the superior stability and solubility of a VHH over a VH. A third distinguishing feature of a VHH is a long CDR3 that can form finger-like extensions and reach cavities on target antigens inaccessible to conventional antibodies. The long CDR3 loop of a VHH often partially folds over the side of the framework corresponding to the side in the VH domain facing the VL. Such folded over loops sterically preclude binding to a VL.

Conventional mAbs are composed of two heavy and two light chains (Figure 1). Both chains contribute to two identical antigen-binding sites. Each target-binding site of a conventional antibody is composed of two non-covalently associated variable domains, designated VH and VL (Figure 2). The target specificity is mediated by three peptide loops at the tip of each V-domain, designated complementarity determining region (CDR). Together, these six CDR loops form the target-binding paratope or idiotype of an antibody. For proper target binding, the two V-domains need to pair up in the proper orientation in order for the CDR loops to jointly form a specific paratope. This is mediated by a hydrophobic interface between the VH and VL domain (illustrated by the black bars in Figures 1 and 2). In intact antibodies, the proper association of VH and VL domains is stabilized by the C-terminal linkage of each V-domain to a constant domain, i.e., CH1 and CL. In most antibodies, these two constant domains in turn are connected by a conserved disulfide bridge which provides further rigidity and stability to the target-binding module.

VH and VL domains can be fused genetically *via* a linker peptide into a small (30 kD) single polypeptide binding module, designated single-chain variable fragment (scFv) (Figure 1). In an scFv, the proper association and orientation of the VH and VL domains to form the target-binding paratope is mediated almost entirely by the hydrophobic interface between the two V-domains. The hydrophobic faces of VH and VL domains can dissociated from one another and associate with other hydrophobic surfaces. This limits the solubility of scFvs and underlies their inherent instability and tendency to aggregate (19).

The VHH domains of camelid heavy chain antibodies have been shaped by more than 50 million years of evolution for high solubility and stability, independent of a partner VL domain. As recombinant proteins, VHH are designated single-domain antibodies or nanobodies (4) in reference to their small size in the nanometer range (20, 21). Importantly, nanobodies have a hydrophilic side (indicated by dashed lines in Figures 1 and 2) corresponding to the light chain interface of VH domains, do not bind light chains, and thus usually do not display any of the solubility and aggregation problems typical of VH domains of conventional antibodies.

A notable difference between the camelid VHH and the human VH domain is the length and orientation of the CDR3 loop (Figure 2). The CDR3 corresponds to the unique region of the antibody molecule that is encoded by a DNA element newly generated during B-cell development. Genetic recombination results in the fusion of a D-element with flanking V- and J-elements. During recombination further genetic diversity is generated by addition and/or deletion of nucleotides at the junctions. Thereby, the CDR3 loop provides the major contribution to antibody diversity and specificity. There is a much lower contribution to diversity by the CDR1 and CDR2 loops, since these loops are germline encoded by a limited number of different V-elements. The CDR3 loop of camelid VHHs shows a much broader distribution of lengths (3–28 amino acids) than human VH domains (8–15 amino acids) (Figure 2) (4, 8). The long CDR3 of a VHH enlarges the potential interaction surface with the target antigen, thereby compensating in part for the missing VL-domain (4, 8). Often, the C-terminal part of a long CDR3 loop folds over onto the side of the VHH domain that corresponds to the side of a VH domain facing a VL domain. This accounts for the often skewed, sideways kind of binding of a VHH domain to its target as compared to the typical head-on binding of a VH–VL pair to its target. This is well illustrated, for example, in the recently reported crystal structure of a PD-L1-specific nanobody with the Ig-domain of PD-L1 and in two of three nanobodies co-crystallized with CD38 (22, 23). The partial folding over of the CDR3 loop onto the former VL interface sterically precludes binding of a VL domain and thereby also contributes to the independence of an nanobody from an associated VL domain. Interestingly, the longer CDR3 of an nanobody can form a finger-like extension that fits into a cavity on the target protein. This allows nanobodies to bind to unique epitopes that are not accessible to conventional mAbs (4, 24, 25), whose antigen-binding interface generally is flat (26).

## Conventional and Heavy Chain Antibodies as Antitumor Therapeutics

The Fc-domain of conventional antibodies can activate the complement system and can serve as recognition module for Fc-receptors on natural killer cells and macrophages. However, the large size of mAbs (150 kDa) is a drawback, as this can limit penetration into tumors *in vivo* (27, 28). It has been estimated that only about 20% of administered mAbs take effect because of their poor pharmacokinetics and weak tissue penetration (29).

In 1993, naturally occurring heavy chain antibodies were discovered serendipitously while analyzing the serum of a dromedary in a practical biochemistry course at the University of Brussels (30). It was soon established that all extant members of the camelid family, i.e., dromedaries, camels, llamas, and alpacas, naturally produce antibodies composed only of heavy chains in addition to conventional antibodies (30, 31). These fully functional antibodies exhibit high specificity, high diversity, and binding capacities similar to those obtained by conventional mAbs, even though they lack the light chain and the CH1 domain of the heavy chain. In camelid hcAbs, a single variable domain, designated VHH, is linked directly to the hinge and Fc-domains of an IgG heavy chain. A heavy chain antibody is, thus, roughly only half the size (75 kDa) of a conventional mAb (150 kDa).

The use of animal-derived antibodies for antitumor therapy is limited, because the immune system typically mounts an antibody response against the foreign components of a therapeutic antibody (32, 33). Neutralization of an antitumor antibody by antibody-specific antibodies generally renders this therapeutic antibody useless for the patient producing anti-antibodies. The constant domains, in particular, are highly immunogenic across species barriers (26, 34, 35). Antibody responses to V-domains are much less frequent. This likely reflects the fact that V-domains undergo extensive somatic hypermutation in every physiological immune response. Somatic hypermutation can result in the substitution of 20 and more amino acid residues in each of the VH and VL domains (36). The human immune system is already tolerized at birth to a huge diversity of V-domains of maternal IgG antibodies that passed the placenta.

**Figure 3:**
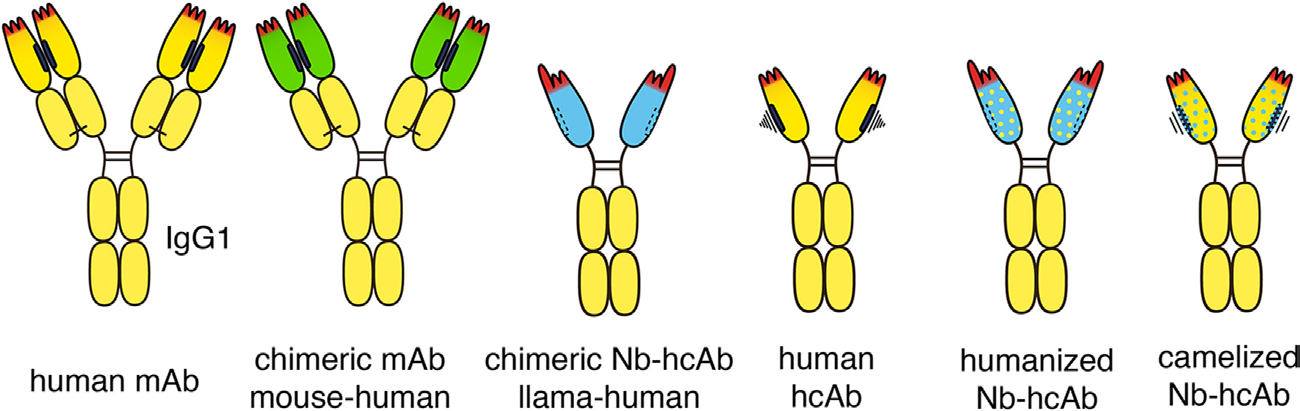
Chimeric and humanized heavy chain antitumor antibodies. Second generation antitumor antibodies such as daratumumab are fully human antibodies, derived from human-Ig transgenic mice or synthetic libraries. Successful antitumor antibodies, such as rituximab and cetuximab, are chimeric antibodies composed of VH and VL domains from mouse monoclonal antibodies (mAbs, green) fused to the constant domains of human IgG1 and kappa, respectively. Chimeric antitumor heavy chain antibodies are easily generated by genetic fusion of a VHH domain (blue) to the hinge and Fc domains of human IgG1. Such chimeric heavy chain antibodies combine the advantageous features of a nanobody (Nb), i.e., high solubility and stability, with the effector functions of a human IgG. Fully human heavy chain antibodies often suffer from the poor solubility and stability of a partnerless VH domain with a vacant sticky hydrophobic side (indicated in black). By substituting divergent framework residues, camelid VHH domains can be “humanized” (yellow dots) and human VH domains can be “camelized” (blue dots) to reduce immunogenicity and to improve solubility, respectively.

Some highly successful antitumor antibodies have been generated simply by replacing the constant domains of the parental mouse antibody with the corresponding domains of human IgG heavy chain and kappa or lambda light chains (Figure 3). Rituximab (anti-CD20) and cetuximab (anti-EGFR), for example, each carry the VH and VL domains of its parental mouse mAb fused to the constant domains of the human IgG1 heavy chain and the human kappa light chain. Analogous chimeric nanobody-human IgG heavy chain antibodies can be generated by fusion of the VHH encoding region to the hinge and Fc domains of a human IgG (37–39).

With respect to immunogenicity, it is important to note that CDR3 loops likely contribute more to immunogenicity than framework residues of the V-domain (26). Indeed, a fraction of patients typically develop anti-idiotype antibodies even against fully humanized antitumor antibodies (33). In such cases, the particular antitumor therapeutic is rendered useless for the patient. As different antibodies against the same target become available, a therapeutic option for these patients will be to switch to a therapeutic antibody with a different idiotype.

VHH domains typically display a high sequence identity with human type 3 VH domains (VH3), likely accounting for their low immunogenicity (40). In addition, humanization of nanobodies can be performed to further minimize their immunogenicity (5, 8, 41). This is accomplished by substituting divergent framework residues with residues commonly found in human VH domains (indicated in Figure 3 schematically by yellow dots). Most divergent residues can indeed be “humanized” without affecting the specificity or solubility of the nanobody-heavy chain antibody. “Humanizing” hydrophilic residues at the side that corresponds to the interface with VL domains, however, can compromise the solubility of the antibody, i.e., render the antibody “sticky” and prone to aggregation.

In order to reduce the residual immunogenicity of animal-derived V-domains in conventional antitumor antibodies, the six CDR loops have been successfully grafted from animal VH and VL domains onto human VH and VL domains (42). Campath-H1 (anti-CD52), for example, contains CDR loops from the parental rat mAb grafted onto the VH and VL domains of human IgG1 kappa (42). The majority of antitumor antibodies currently in clinical development are derived from fully human antibodies (43). Daratumumab (anti-CD38), for example, was generated from immunized human Ig-transgenic mice, necitumumab (anti-EGFR) was selected from a fully synthetic Fab display library (44).

Attempts have also been made to generate fully human heavy chain antibodies (45) (Figure 3). Such fully human heavy chain antibodies, however, suffer from poor solubility, likely due to the inherent tendency of the non-paired VH domain to bind to free light chains and to aggregate. Solubility of human VH domains can be improved by “camelization,” e.g., by substituting residues in the hydrophobic interface with hydrophilic residues (indicated in Figure 3 schematically by blue dots) (46). This can ameliorate the aggregation and stickiness of human heavy chain antibodies. Further preclinical and clinical studies will show whether heavy chain antibodies based on “camelized” VH domains or on “humanized” VHH domains are better suited as antitumor therapeutics.

## Bispecific Antitumor Heavy Chain Antibodies

**Figure 4:**
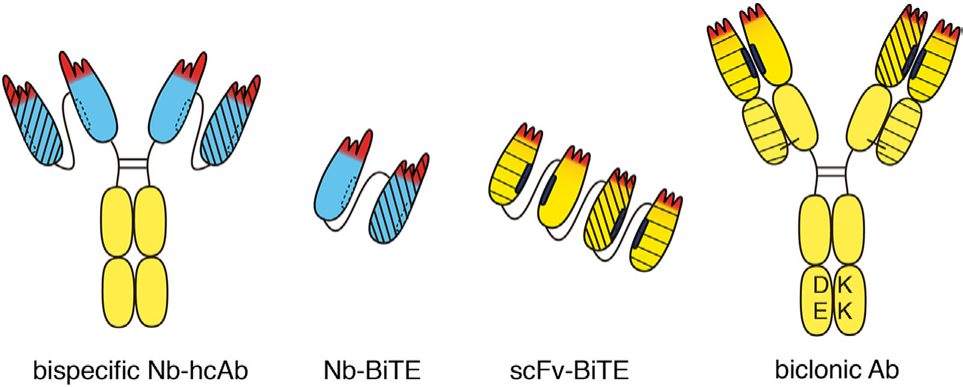
Bispecific heavy chain antibodies. A bispecific heavy chain antibody can be generated simply by linking two tandem VHH domains (blue) to the hinge and Fc domains of human IgG. Both specificities are contained in a single heavy chain. Therefore, there is no need to engineer the Fc-domain as in conventional biclonics. Two nanobodies with different specificities can be linked to one another without any mispairing issues to generate a bispecific construct (Nb-BiTE). Two different single-chain variable fragment (scFv) domains can be linked genetically to one another, e.g., to generate a bispecific T-cell engager (BiTE). Special precautions are required to prevent mispairing of VH and VL domains. Moreover, the hydrophobic interface of VH and VL domains can dissociate from one another and associate with other hydrophobic surfaces. This can severely limit the stability and solubility of scFv modules. Bispecific antibodies can be generated from two distinct heavy chains. In order to circumvent mispairing of VH–VL domains, a common “fixed” light chain is used that pairs with both heavy chains. In this case, target specificity is mediated largely if not entirely by the two VH domains. Fc-engineering is commonly used to favor formation of heteromeric over homomeric antibodies, e.g., by electrostatic steering to introduce negatively charged amino acid residues (DE) in one CH3 domain and matching basic residues (KK) in the other CH3 domain. Different line patterns are used to indicate different specificities of VHHs and VHs.

Often, the target antigen of an antitumor antibody is expressed not only by tumor cells but also by healthy cells. In such cases, the target on healthy cells can act as a sink for the therapeutic. Cytotoxicity of the antitumor antibody to healthy cells can cause unwanted side effects. One strategy to improve the specificity of antitumor antibodies is to genetically link the target-binding modules of two distinct tumor-targeting antibodies into a single, bispecific antibody (bsAb) (47, 48) (Figure 4). The halves of two conventional antibodies can be combined to generate a bsAb. Catumaxumab, for example, contains an EpCAM-specific “half” antibody connected *via* disulfide bonds in the hinge region to a CD3-specific “half-mAb” (49). In this case, usage of antibodies from two distinct species, e.g., rat IgG2a and rat lambda on the one side and mouse IgG2b and mouse kappa on the other side, reduces mispairing of the VL and VH domains (50). When generating a bsAb from two distinct human mAbs, genetic engineering is usually required to ensure proper pairing of the two VH and VL domains. One strategy employs a common “fixed” light chain that can pair with both heavy chains. In this case, target specificity is mediated by the VH domains, while the common VL domain contributes only little if anything to target binding. Additional engineering—e.g., electrostratic steering or insertion of a “knob” in one CH3 domain and a “hole” in the other CH3 domain—is often used to favor heteromeric over homomeric pairing of heavy chains (51). For example, the biclonic MCLA-128 (anti-HER2—anti HER3) contains a fixed human IgV kappa chain, and DE-KK Fc-engineered heavy chains (L351D L368E, L351K T366K) (52).

Since nanobodies have a completely hydrophilic surface and do not bind light chains, they can be easily linked into dimers and multimers without need for additional measures. Thus, a bispecific nanobody-based heavy chain antibody can be generated simply by genetically fusing a second nanobody *via* a linker peptide to the N-terminus of a heavy chain antibody (Figure 4). Since both specificities are contained in a single polypeptide in bispecific heavy chain antibodies, there is no need to engineer the Fc-domain as in conventional biclonics. Such nanobody-based bispecific heavy chain antibodies routinely show excellent solubility, stability, and production yields akin to their parental heavy chain antibodies (our own unpublished observations).

A similar strategy, i.e., the genetic linkage of two target-binding modules, can also be used to attract and link mobile immune cells to cancer cells, e.g., with one of the modules binding to a tumor cell and the other to a T cell or an NK cell (53). For linking T cells or NK cells to tumor cells, the Fc domain is dispensable. Thus, two scFv domains can be linked genetically to one another, e.g., to generate a bispecific T cell engager (BiTE) or bispecific NK-cell engager (BiKE) (Figure 4). Blinatumumab, for example, contains a CD19-specific scFv linked to a CD3-specific scFv (54). When linking two or more scFvs, however, special precautions are required to prevent mispairing of VH and VL domains (55). In contrast, nanobodies can readily be fused into BiTEs or BiKEs with little, if any, solubility or stability issues.

Dimerization of two nanobodies or scFvs can also be achieved by genetic fusion to natural or synthetic dimerization domains (56). Fusion to the upper hinge region, for example, allows dimerization *via* formation of interchain disulfide bonds between cysteine residues in the hinge (57). Heterodimer-formation can be forced by fusion of two nanobodies or scFvs to distinct dimerization peptides or protein domains, e.g., fos-jun leucine zippers (58) or two distinct CH3 domains carrying electrostatic steering modules (L351D L368E, L351K T366K) (52) (as in Figure 4) or a hydrophobic “knob” (T366W) and a matching “hole” (T366S, L368A, and Y407V), respectively (51). In the context of tumor therapy, such nanobody dimers can be used as BiTEs or BiKEs to enhance the binding of cytotoxic lymphocytes to tumor cells.

## Nanobodies as Antitumor Therapeutics

Antitumor therapeutics require a homogenous distribution within the entire tumor for successful tumor treatment. If only part of the tumor is exposed to the therapeutic, complete tumor eradication will not be achieved, leading eventually to tumor regrowth (14). In this regard, nanobodies are expected to outperform mAbs due to their small size and good tumor penetration *in vivo* (28, 59, 60). Nanobodies can readily be cloned into various formats by fusion to other proteins or effector domains, thereby tailoring their utility for specific therapeutic applications.

Antitumor nanobodies can be categorized into three types: naked monomeric or multimeric nanobodies, nanobodies genetically fused to effector domains, and as targeting moieties on liposomes or nanoparticles encapsulating a drug (10, 14). Below we describe and discuss the use of antitumor nanobodies according to these basic concepts.

## “Naked” Monomeric and Multimeric Antitumor Nanobodies

**Figure 5:**
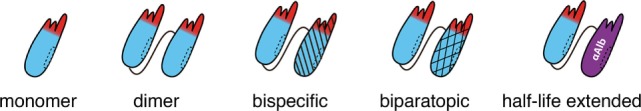
Schematic representation of di- and multimeric antitumor nanobodies. Owing to their high solubility and stability nanobodies can readily be fused genetically to other nanobodies without the mispairing and solubility issues inherent to single-chain variable fragment-based dimers and multimers. Flexible glycine–serine linkers are commonly used to fuse nanobodies, e.g., one or more tandem modules of G4S composed of four glycine residues to provide maximal flexibility and a hydrophilic serine residue to improve solubility. Tandem fusion of two identical nanobodies yields a bivalent dimer, often with improved avidity over the respective monomer. Tandem cloning of two distinct nanobodies that recognize non-overlapping epitopes of the same antigen yields a biparatopic binder. Fusion of two nanobodies recognizing distinct cell surface proteins yields a bispecific binder. The *in vivo* half-life can be extended by fusing one or more antitumor nanobodies to an albumin-specific nanobody. Piggy-backing on albumin reduces the loss of antitumor nanobodies by renal filtration.

In oncology, “naked” nanobodies without a linked Fc domain have many interesting potential applications. Their high thermal stability, high refolding capacity, and good tissue penetration *in vivo* (28, 57, 61, 62) make nanobodies ideally suited for specific and efficient targeting of tumor antigens *in vivo*. Because of the modular and single-domain characteristic of nanobodies, molecular manipulation for generating multivalent or multispecific single-chain antibody molecules is relatively easy (Figure 5). Tandem cloning of two identical nanobodies connected by a linker peptide, e.g., a flexible glycine–serine linker, yields a bivalent molecule (30–35 kDa) with higher avidity for the antigen (57, 63). Similarly, tandem cloning of nanobodies that recognize two different epitopes of the same antigen yields a biparatopic molecule. The improved avidity of bivalent nanobodies leads to a reduced off-rate and a reduced release of the nanobody reagent from its target. Crosslinking of a target by a nanobody dimer can induce apoptosis and other signaling cascades (64) or internalization of the target molecule (65).

Fusion of an nanobody monomer or dimer to an albumin-specific nanobody increases the *in vivo* half-life of the reagent (64, 66–68). This is an elegant strategy to overcome the inherent disadvantage of the small size of nanobodies in the context of antitumor therapy *in vivo*. The size of monomeric, dimeric, and trimeric nanobodies is below the renal filtration sieve (approximately 60 kD). Thus, unbound nanobodies are rapidly cleared from the bloodstream by renal elimination. The resulting short *in vivo* half-life of 1–2 h reduces the time interval to bind to their target molecule within the tumor (28, 69, 70). Caplacizumab, the first nanobody expected to be licensed for clinical use in 2018, consists of a dimeric nanobody (directed against von Willebrand factor) (71). A downside of dimeric and trimeric nanobodies is an increased size (30 and 45 kDa, respectively), resulting in a less efficient tumor penetration compared to monovalent nanobodies (15 kDa) (72).

In the context of cancer therapy, nanobodies have been developed against growth factor receptors and their ligands, chemokine receptors, death receptors (DR), and ecto-enzymes. Nanobodies against “classical” receptor targets can antagonize ligand binding and activation of signaling cascades by targeted tumor cells (14). For example, nanobodies against epidermal growth factor receptor (EGFR) (25, 64, 73–75), hepatocyte growth factor receptor (HGFR, c-Met) (76), human epidermal growth factor (HER2) (77, 78), and VEGFR (79) inhibit signaling by their respective ligands. Recently, new potential antitumor nanobodies have been developed against other membrane protein targets such as the DRs DR5 (63, 80) and survivin (81), the chemokine receptors CXCR4 (82) and CXCR7 (83), the ion channel P2X7 (84), and the ecto-enzyme CD38 (24, 85). Alternatively, antitumor nanobodies can be directed against receptor ligands, such as HGF (68), VEGF (86, 87), urokinase-type plasminogen activator (88), or CXCL11/12 (89). In a number of *in vivo* xenograft studies, treatment with bispecific or multivalent nanobodies resulted in delay of tumor growth (64, 68) and/or inhibition of angiogenesis (83). An example of a half-life extended antitumor nanobody is CONAN-1, a biparatopic anti-EGFR nanobody fused to an anti-albumin nanobody (64). Fusion to the anti-albumin nanobody increased the half-life of the antitumor nanobody from 1–2 h to 2–3 days. Importantly, in an *in vivo* model of athymic mice bearing tumor xenografts, CONAN-1 inhibited tumor outgrowth with a similar potency as the conventional mAb cetuximab, despite the fact that CONAN-1 is devoid of an Fc-domain that could mediate immune effector functions. CONAN-1 was also more potent than bivalent, monospecific nanobodies in inhibiting tumor growth (64). A recent study demonstrated the potential advantage of a bi-functional molecule, comprising an EGFR-targeted nanobody and DR-targeted ligand TRAIL (90). The results revealed that ENb-TRAIL has therapeutic efficacy in different tumor entities, which do not respond to either EGFR antagonist or DR agonist monotherapies.

## Nanobodies as Targeting Moieties of Effector Domains

**Figure 6:**
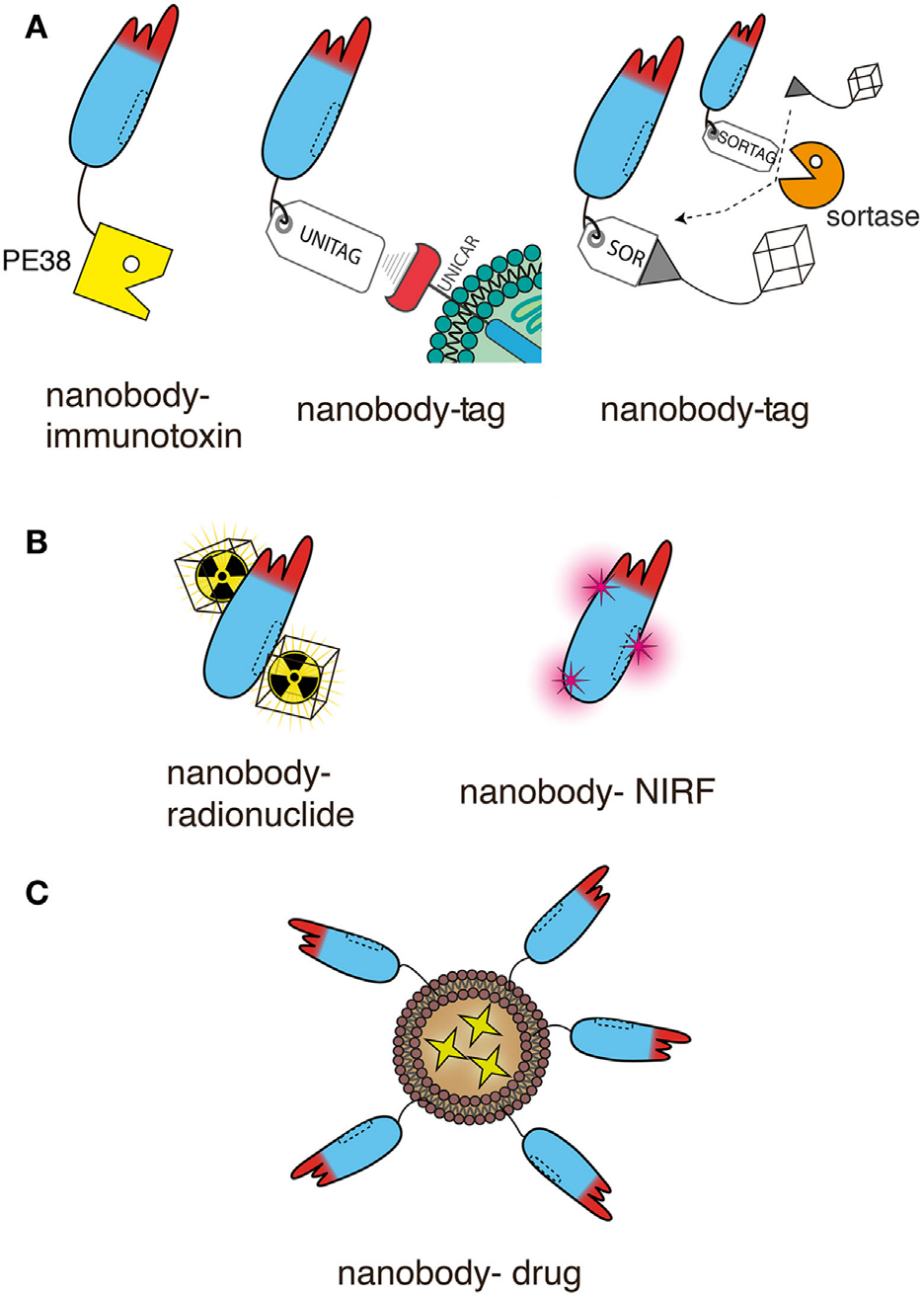
Schematic representation of nanobodies as targeting moieties of effector domains for antitumor therapy. The schematics illustrate genetic fusion **(A)** and chemical conjugation **(B,C)** of nanobodies to effectors. For simplicity only a single nanobody is shown in each schematic. This nanobody can readily be replaced by any of the dimeric and multimeric constructs shown in Figure 5, usually without compromising solubility. Moreover, the unitag and sortag technologies can be used also with the Nb-hcAbs illustrated in Figures 2–4. **(A)** Nanobodies can be fused genetically to other proteins and/or to smaller peptides. Both, the N- and C-terminus are available for fusion. The diagrams illustrate the more commonly used C-terminus. Fusion to a toxin such as *Pseudomonas* exotoxin A (PE38) generates an immunotoxin. Fusion to a peptide tag provides a means to deliver a universal marker onto tumor cells, e.g., as a docking site for a universal tag-specific antibody or T cell transduced with a tag-specific chimeric antigen receptor. Fusion to a sortag provides a substrate for site-specific, sortase-catalyzed linkage to a synthetic peptide, e.g., GGGX. When X = lysine or cystein, almost any chemical moiety can be conjugated by amide or maleimide chemistry, including a chelator for a radionuclide. **(B)** Nanobodies can also be conjugated chemically to the side chains of lysine or cysteine residue. Amide conjugation usually introduces multiple modifications, since nanobodies typically contain several surface-exposed lysine residues and an N-terminal amine. Since most nanobodies contain only two deeply buried cysteine residues engaged in an intrachain disulfide bond, malemeide conjugation requires the introduction of one or more surface exposed cysteine residues by site directed mutagenesis. These techniques allow conjugation of radionuclides or near-infrared fluorochromes (NIRFs). **(C)** Nanobodies can be easily linked, e.g., *via* a C-terminal polyethyleneglycol moiety, to nanoparticles, such as liposomes containing anti-neoplastic drugs.

The antitumor effects of nanobodies can be enhanced by coupling nanobodies to protein, peptide, and chemical effectors (Figure 6). Effector-equipped nanobodies combine the advantages of high specificity of the nanobodies, their potential intrinsic therapeutic effects as antagonists, good tissue penetration, and specific accumulation within tumors, with the cytotoxic effects mediated by the effector. Lack of an Fc-domain may be advantageous in such cases, as Fc-mediated clearance may diminish delivery of the effector domain to the tumor (16). However, the attachment of foreign proteins, peptides, and chemicals also introduces potentially immunogenic epitopes and leads to an increase in size which may reduce the efficiency of penetration into the tumor (72).

Genetic fusion and chemical conjugation provide two, partially overlapping, options for equipping nanobodies with effectors. The following sections describe the combination of nanobodies with different effectors, including genetic fusion of nanobodies to protein toxins and peptides, and chemical conjugation to radionuclides, photosensitizers, and anti-neoplastic drugs.

## Nanobody-Based Immunotoxins

Immunotoxins consist of a targeting moiety linked to a toxin and are designed to specifically kill targeted tumor cells. The toxin moiety (*Pseudomonas* exotoxin, *Diphtheria* toxin, ricin, cucurmosin) induces cell death, while the targeting moiety binds to antigens preferentially expressed on the cancer cell surface, minimizing cytotoxic side effects on normal cells. Nanobody-based immunotoxins typically are generated by genetic fusion of the protein toxin to the C-terminus of the nanobody, i.e., akin to fusion of an nanobody to the hinge and Fc domains described above (Figure 6A). For example, a dimeric anti-VEGFR2 nanobody fused to the truncated form of *Pseudomonas* exotoxin A (PE38) effectively inhibited the proliferation of VEGFR2-expressing cells *in vitro* (79). A similar fusion construct of a monomeric CD38-specific nanobody with PE38 resulted in highly selective cytotoxicity against multiple myeloma cell lines and patient-derived multiple myeloma cells (23). And CD7-specific nanobodies fused to PE38 showed cytotoxic efficacy in CD7-expressing T-cell acute lymphoblastic leukemia *in vitro* and in a preclinical mouse model *in vivo* (91, 92). Genetic fusion of an anti-EGFR nanobody to cucurmosin, a pumpkin toxin from the family of type 1 ribosome inactivating proteins induced cell death of EGFR-expressing cells lines *in vitro* (93).

A drawback of nanobody-based immunotoxins is the inherent immunogenicity of the foreign protein toxin (94). As in case of animal-derived constant Ig domains, the human immune system usually mounts a strong antibody response to the protein toxin. Consequently, nanobody-based immunotoxins should be considered for single use only, at least until reproducible tolerization strategies have been established. Immunotoxins, thus, represent targeted antitumor therapeutics, in particular for cancer patients in which standard treatment is no longer an option (95).

## Nanobody-Peptide Fusions

C-terminal fusion of nanobodies to short peptide-tags is commonly used as a tool to facilitate the purification and detection of bound nanobodies, e.g., *via* a tag-specific antibody (Figure 6A). Peptide-tagged nanobodies can also be used to mark tumor cells for attack by tag-specific T cells (96). Peptide-tagging further provides an elegant means for site-specific conjugation of nanobodies to any chemical moiety (97).

Fusion of an antitumor nanobody to a peptide tag provides a tool to deliver a universal marker onto the tumor cells, e.g., as a docking site for a universal cytotoxic tag-specific antibody or for T cells transfected with a tag-specific chimeric antigen receptor (CAR). This has been demonstrated recently in an elegant proof of principle study with an EGFR-specific nanobody fused to the E5B9 peptide tag (the UniTag) (96). Opsonization of EGFR-expressing tumor cells with the nanobody-tag rendered the tumor cells highly sensitive for attack by human peripheral blood T cells that had been transduced to express a tag-specific CAR (UniCAR T cells).

Peptide-tagging can also be used as a tool for site-specific, enzyme catalyzed conjugation of an nanobody to virtually any desired chemical compound (Figure 6A). Genetic fusion of an nanobody to a C-terminal pentapeptide (Sortag) provides a substrate for site-specific, sortase-catalyzed C-terminal linkage of the nanobody to a small synthetic peptide, e.g., GGGX (97–100). “X” can be a lysine or cysteine residue conjugated by amide or maleimide chemistry to virtually any chemical moiety, e.g., a near-infrared fluorochrome (NIRF), a poly-ethylene-glycol tail, a chelator for a radionuclide, or tetrazine as a basis for click-reactions, e.g., for simple attachment of radioisotopes for PET imaging (97, 99, 100). In an elegant proof of principle study, sortagging was used to image tumor-infiltrating macrophages using ^18^F-labeled CD11c-specific nanobodies (97). The same group has recently reported the use of sortagging to attach a bi-functional tag to the C-terminus of a CD8-specific nanobody for imaging of tumor-infiltrating cytotoxic T cells (101). A chelator was used to install ^89^Zr for PET imaging and an azide functionality for PEGylation. ^89^Zr provided crisp PET images of lympoid organs and CTL-infiltrated tumors, a 20-kD PEG moiety provide a much reduced accumulation of the labeled nanobody in the kidney compared to non-PEGylated nanobodies.

Akin to sortase-catalyzed transpeptidation of an nanobody fused to a sortag, BirA-catalyzed biotinylation of a specific peptide tag (Avi-tag) can be used to site-specifically biotinylate nanobodies carrying an Avi-tag (102). BirA-catalyzed biotinylation can be achieved both *in vitro* with purified BirA or in cells by co-expression of BirA in the same cellular compartment as the Avi-tagged nanobody (103, 104). Biotinylation provides a universal anchor for high-affinity binding to Streptavidin-conjugates.

## Nanobody-Targeted Radionuclides and NIRFs

Nanobodies can readily be conjugated to radionuclides and fluorochromes, using either sortagging or classical chemical conjugation strategies (Figure 6B). Such nanobody-radionuclide and nanobody-NIRF conjugates are useful tools for imaging of tumors antigens or tumor-associated stromal cells, such as the mannose receptor of macrophages (MMR, CD206) (11, 16, 105–107). Moreover, such conjugates also have therapeutic potential, e.g., by local delivery of ionizing radiation to the tumor or by thermal cytotoxicity *via* a photosensitive, NIRF.

Conventional protein conjugation strategies use random conjugation to reactive side chains, most commonly amide conjugation to the amino group of lysine side chains or to the N-terminus of the protein (which, in case of V-domains, lies in proximity to the antigen-binding paratope). Random conjugation is difficult to control and may compromise the functionality of the nanobody, e.g., by sterically interfering with target binding, by sterically compromising tissue distribution, and by providing potentially immunogenic epitopes. Two elegant approaches have been developed to conjugate chemicals to nanobodies at a specific site. One involves the introduction of a cysteine residue at the C-terminus or at specific framework residues, providing a basis for site-specific maleimide conjugations (99, 102). The other method introduces a pentapeptide (LPXTG) that allows sortase-catalyzed transpeptidation (100) (see above).

Targeted radionuclide therapy is a systemic treatment that aims to deliver cytotoxic radiation to cancer cells and to cause at the same time minimal toxicity to surrounding healthy tissues. Radiopharmaceuticals consist of two components: a targeting moiety that specifically determines the accumulation of the radiopharmaceutical in the tumor and a radionuclide that delivers cytotoxic radiation through its decay (15). There is a growing interest in the use of nanobodies as targeting moieties for targeted radionuclide therapy (Figure 6B) (11). Nanobodies represent ideal candidates due to their high stability in harsh conditions, such as elevated temperatures and extreme pHs, offering the advantage to use a broader range of radiochemistry methods (15).

The utility of nanobodies as vehicles for targeted radionuclide therapy has been investigated in several preclinical models. An *in vivo* study demonstrated that ^177^Lu-labeled anti-HER2 nanobodies efficiently targeted HER2-positive xenografts and prevented tumor growth, while keeping radioactivity levels low in normal organs (108). Another preclinical study in mice demonstrated that ^177^Lu-labeled anti-idiotype nanobodies led to an inhibition of disease progression in multiple myeloma (109). However, radiolabeled nanobodies are characterized by fast clearance through kidneys, resulting in suboptimal absolute tumor uptake but intense renal accumulation. Nephrotoxicity may be reduced by coadministration of gelofusin and lysine. This has been shown to reduce renal uptake of a ^99^mTc-labeled anti-EGFR nanobody by 45% in tumor xenografted mice (110).

Taken together, radiolabeled nanobodies are promising targeting moieties for targeted radionuclide therapy. Nanobody-based radionuclide therapy may be particularly beneficial in the treatment of micrometastatic and minimal residual disease, due to a highly specific deposition of radioactivity to tumor cells.

Photodynamic therapy induces cell death through light activation of a photosensitizer. NIRFs such as IRDye700DX can function as traceable photosensitizer (65, 100). For example, an EGFR-specific nanobody-photosensitizer conjugate rendered tumor cells sensitive to light induced death *in vitro* and in an orthotopic mouse tumor model *in vivo* (111).

## Nanobody-Targeted Nanoparticles

Another approach for specific drug delivery is the generation of targeted nanoparticles (<200 nm), as encapsulation of drugs overcomes problems, such as poor solubility, limited stability, and rapid clearance (16) (Figure 6C). Nanoparticles used for this approach include liposomes (112, 113), micelles (114, 115), albumin-based nanoparticles (116, 117), and polymer-based polymersomes (118) or polyplexes (119). Nanobodies are advantageous for the decoration of the surface of nanoparticles due to their small size and the absence of an Fc-domain, as it decreases the chance of immunogenic responses and delay the clearance of these nanobody-targeted nanoparticles (120). *In vitro* experiments employing nanobodies as targeting moieties of nanoparticles have shown improved binding to the target cells (112, 115, 117, 121). *In vivo*, nanoparticles do not effectively cross endothelial barriers. It has been proposed that accumulation of the targeted nanoparticles within tumors is facilitated by the enhanced permeability and retention effect. The abnormal structure of rapidly growing tumor vasculature, combined with the lack of proper lymphatic drainage, leads to the accumulation of nanoparticles (122, 123). In cases where endothelial cells of the tumor vasculature express specific cell surface markers, it is feasible to specifically address nanoparticles to the tumor vasculature.

Release of the drugs from the particles can be achieved by leakage or by mechanical destruction by ultrasound or intracellular degradation. The first liposomes that were decorated with anti-EGFR nanobodies were internalized into the target cell (112). Anti-EGFR nanobody-targeted polymeric micelles containing doxorubicin were significantly more effective at inhibiting tumor growth and prolonging the survival of animals compared with untargeted micelles (114).

## Viral and Cellular Delivery of Antitumor Nanobodies and Heavy Chain Antibodies

While size does matter, it is not the only factor determining the delivery of nanobody-based biologics to tumor cells. Tumor delivery is controlled by a complex interplay of factors, many of which are still poorly understood: the site of injection (e.g., intravenous, subcutaneous, intratumor), transport *via* the blood and lymphatics, diffusion through the endothelial cell and basement membrane into the interstitial space, hydrodynamic pressure in the blood vs. the tumor tissues, elimination of biologics from the system (e.g., by renal filtration, hepatic excretion, endocytosis by cells), binding to non-tumor cells, and binding to proteins (e.g., albumin, rheumatoid factor, other preformed or induced antibodies). It may, therefore, be of interest to consider and test other options for the delivery of antitumor nanobodies and heavy chain antibodies.

One interesting option is to use circulating cells of the immune system for antibody delivery. Immune cells are not or less effected than proteins by hydrodynamic pressure, endothelial barriers, and renal or hepatic excretion. Cells can effectively migrate through endothelial barriers and into the tumor microenvironment. Cells are eliminated by apoptosis and phagocytosis rather than by renal or hepatic excretion. A potentially powerful technique is to transduce T cells or NK cells to express a CAR on the cell surface (124, 125). CARs typically contain a scFv linked *via* a transmembrane domain to cytosolic activation domains. In nanobody-based CARs, the relatively unstable scFv is simply replaced by a stable nanobody (126–129). CAR-expressing T cells and NK cells can serially bind and kill many tumor cells expressing the target antigen. It is also conceivable that immune cells can be similarly transduced to secrete antitumor nanobodies, Nb-hcAbs, and other nanobody-based biologics.

Adeno-associated viruses (AAV) have been used successfully as gene-therapy vectors, i.e., for the long-term expression of a therapeutic proteins *in vivo* (130, 131). For example, intramuscular injection of AAV encoding HIV-neutralizing antibodies or a CD4–Fc fusion protein led to long-term production of the encoded antibodies and protection of mice from HIV infection (131, 132). In a recent proof of principle study, an AAV-encoded bispecific nanobody was effectively expressed *in vivo* and exhibited therapeutic efficacy in a mouse model of amyloidosis (133). It is conceivable that AAV can similarly be engineered for local and/or long-term expression of antitumor nanobodies *in vivo*.

## Conclusion and Perspectives

Nanobodies, nanobody-based heavy chain antibodies, and nanobody-drug conjugates have a huge potential as antitumor therapeutics. The US Food and Drug Administration recently granted fast track designation for caplacizumab, a bivalent nanobody targeting von Willebrand factor. Ablynx, the leading nanobody biotech company, has submitted an application for European Marketing Authorisation for caplacizumab, which thus may well become the first nanobody approved for therapy. Before nanobody-based antitumor therapeutics follow suite, additional preclinical and clinical studies are warranted. Antitumor nanobodies and antitumor Nb-hcAbs may overcome some of the obstacles that hamper therapies with antitumor mAbs. *In vivo* studies have underscored the favorable biodistribution of nanobodies, including deep penetration into tumors. Numerous nanobody-based biologics have shown antitumor efficacy in preclinical studies *in vivo*. Naked nanobodies can antagonize growth factor receptors and block ion channels and ecto-enzymes in the tumor microenvironment. Fusion of one or more nanobodies to the hinge and Fc-domains of a human immunoglobulin yields highly soluble and versatile heavy chain antibodies. Importantly, because nanobodies do not bind light chains and because they do not show any tendency to aggregate, nanobody-based bispecific hcAbs do not suffer from the VH–VL pairing problem of bispecifc conventional antibodies. Heavy chain antibodies are roughly half the size of conventional antibodies and, thus, may show better tissue penetration than conventional mAbs, while retaining the capacity to recruit the complement system, NK cells, and macrophages. Genetic fusion of nanobodies to peptide tags opens a path for marking tumor cells for attack by tag-specific T cells or NK cells transduced with a tag-specific CAR or with universal tag-specific cytotoxic antibodies. Sortagging and introduction of cysteine residues at specific framework positions allow easy conjugation to virtually any chemical moiety, including chelators for radionuclides, NIRFs, polyethylene glycol, liposomes, and nanoparticles. Preliminary studies indicate that it may well be worthwhile to further explore other modes for effective targeting of tumor cells with antitumor nanobodies, including Nb-CAR-expressing T cells and NK-cells and AAV encoding antitumor nanobodies.

V-domains display a much lower intrinsic immunogenicity than Fc domains across species barriers. Moreover, nanobodies show higher sequence identity to human VH domains than do murine VH domains and divergent framework residues routinely are “humanized” in clinical nanobodies. While fusion to the hinge and Fc domains of human IgG does not add any additional immunogenicity, fusion to toxins, peptide tags, and chemical conjugation add potentially immunogenic epitopes. Moreover, as with conventional antibodies, the antibody paratope can induce an anti-idiotypic antibody response in a fraction of patients. As distinct nanobodies to the same target protein become available, switching to a different antitumor nanobody will become a therapeutic option. Assuming that progress will continue at the present pace, it is likely that the future repertoire of clinicians will include an increasing battery of nanobody-based antitumor therapeutics.

## Author Contributions

FK-N and PB conceived the topic; PB, JH, and FK-N wrote the manuscript.

## Conflict of Interest Statement

PB and FK-N are coinventors on a patent application on CD38-specific nanobodies. FK-N receives a share of antibody and protein sales *via* MediGate GmbH, a wholly owned subsidiary of the University Medical Center Hamburg-Eppendorf.
